# Quaternary Benzophenanthridine Alkaloids Act as Smac Mimetics and Overcome Resistance to Apoptosis

**DOI:** 10.3390/ijms242015405

**Published:** 2023-10-20

**Authors:** Petra Kulíšková, Lucie Vašátková, Iva Slaninová

**Affiliations:** 1 Department of Biology, Faculty of Medicine, Masaryk University, Kamenice 5, Building A6, 62500 Brno, Czech Republic; 461133@mail.muni.cz (P.K.); supolikova@med.muni.cz (L.V.); 2 Department of Clinical Immunology and Allergology, Faculty of Medicine, Masaryk University, 62500 Brno, Czech Republic

**Keywords:** apoptosis, benzophenanthridine alkaloids, cancer, cell death, cIAP, Smac mimetic drug resistance, chelerythrine, sanguinarine

## Abstract

Defects in cell death signaling pathways are one of the hallmarks of cancer and can lead to resistance to conventional therapy. Natural products are promising compounds that can overcome this resistance. In the present study we studied the effect of six quaternary benzophenanthridine alkaloids (QBAs), sanguinarine, chelerythrine, sanguirubine, chelirubine, sanguilutine, and chelilutine, on Jurkat leukemia cells, WT, and cell death deficient lines derived from them, CASP3/7/6^-/-^ and FADD^-/-^, and on solid tumor, human malignant melanoma, A375 cells. We demonstrated the ability of QBAs to overcome the resistance of these deficient cells and identified a novel mechanism for their action. Sanguinarine and sanguirubine completely and chelerythrine, sanguilutine, and chelilutine partially overcame the resistance of CASP3/7/6^-/-^ and FADD^-/-^ cells. By detection of cPARP, a marker of apoptosis, and pMLKL, a marker of necroptosis, we proved the ability of QBAs to induce both these cell deaths (bimodal cell death) with apoptosis preceding necroptosis. We identified the new mechanism of the cell death induction by QBAs, the downregulation of the apoptosis inhibitors cIAP1 and cIAP2, i.e., an effect similar to that of Smac mimetics.

## 1. Introduction

Resistance of cancer cells to therapy represents a major obstacle in clinical oncology. Defects in programmed cell death signaling pathways are one of the causes of multidrug resistance.

One possibility to facilitate the induction of apoptosis in resistant cells is the use of cIAP inhibitors, Smac mimetics (SMs). Smac (second mitochondrial activator of caspases) protein releases from mitochondria after apoptotic signaling and by the inhibition of cIAPs (cellular inhibitor of apoptosis proteins) augment apoptosis induction. SMs are small molecular inhibitors used as anti-cancer drugs [1]. Birinapant and LCL161 are SMs used for example in the therapy of multiple myeloma [2], glioblastoma [3], and sarcoma [4]. cIAP proteins are components of signaling molecular complexes that regulate cell fate, such as whether a cell undergoes cell death by apoptosis or necroptosis, or whether it survives (NF-κB signaling). cIAPs function also as E3 ubiquitin ligases for the RIP1 kinase, a key player in necroptosis. Depending on its ubiquitination status, RIP1 either promotes survival by stimulating NF-κB activation once it is ubiquitinated, or contributes to cell death in its deubiquitinated form, which allows its interaction with key components of death receptor signaling such as FADD and caspase-8 [5]. cIAP1 and cIAP2 proteins are expressed at high levels in many malignancies including leukemia, and contribute to the failure of apoptosis [6]. Thus, IAPs represent promising targets for the development of new biomarkers and cancer therapeutics in hematological and other malignancies. Petersen et al. [7] reported that SMs primarily kill cancer cells also through autoubiquitination and degradation of cIAPs, which results in TNF-α-mediated cell death. New compounds that act like SM could help overcome the resistance of cancer cells to apoptosis.

Quaternary benzo[c]phenanthridine alkaloids (QBAs) are natural products, isochinoline alkaloids, which occur in many plant species of the families *Papaveraceae*, *Fumariaceae*, *Ranunculaceae*, and *Rutaceae*. Herbal extracts from these plants are often used in traditional Chinese medicine for their antifungal, antimicrobial, and anti-inflammatory effects [8]. Only SA and CHE are commercially available and their effects on cancer cells, including apoptosis induction, have been described and reviewed by many authors [9,10,11,12]. Although QBAs are small molecules, their effect is multitarget. Due to quaternary nitrogen, which is highly reactive, alkaloids interact with various proteins and enzymes. Moreover, their molecule is planar and can interact with DNA. Both SA and CHE have been shown to interact with DNA G-quadruplexes [13], which explains their effect on the expression of some genes and their anti-telomerase activity. CHE was identified as an inhibitor of PKC [14], a molecule involved in the regulation of important signaling pathways including apoptosis and multidrug resistance. SA also induces apoptosis through numerous pathways, including the activation of NF-κB [15], the mitochondrial damage resulting in the activation of the caspase machinery [16], and cell cycle arrest [17]. SA and CHE also downregulate Bcl-2 or Bcl-XL anti-apoptotic proteins [18,19,20,21]. Minor QBAs sanguilutine (SL), chelilutine (CHL), sanguirubine (SR) chelirubine (CHR), and macarpine were studied to a lesser extent mostly by our group [21,22,23,24,25].

Here, we demonstrate the ability of QBAs to overcome drug resistance and induce cell death of cancer cells with defects in apoptotic pathways. We identified a novel mechanism of QBA action, the induction of bimodal cell death by affecting levels of antiapoptotic cIAP1 and cIAP2 proteins.

## 2. Results

### 2.1. QBAs Induce Cell Death in Apoptosis-Resistant Cells

In our previous study, we demonstrated the resistance of Jurkat cells deficient in FADD and/or executioner caspases (FADD^-/-^ and CASP3/7/6^-/-^ cells) on common anti-cancer drugs. We were interested to know if any compounds could overcome this resistance. Therefore, we studied the effect of natural products, benzophenanthridine alkaloids, sanguinarine (SA, 1 µg/mL), chelerythrine (CHE, 1 µg/mL), sanguilutine (SL, 1 µg/mL), chelilutine (CHL, 1 and 2 µg/mL), sanguirubine (SR, 1 and 2 µg/mL), and chelirubine (CHR, 1 and 2 µg/mL) on cells viability using the PI exclusion assay. Doxorubicin (Doxo) was used as a control anti-cancer drug.

**Figure 1:**
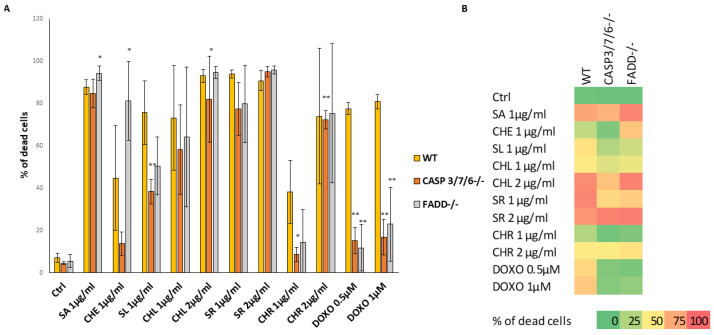
Cytotoxicity of QBAs on Jurkat cells. (**A**) Flow cytometry analysis of propidium iodide stained Jurkat cells showing the percentage of dead cells after 48 h treatment with sanguinarine (SA), chelerythrine (CHE), sanguirubine (SR), chelirubine (CHR), sanguilutine (SL), and chelilutine (CHL). Doxorubucine (DOXO) was used as a control anti-cancer drug. Data are means ± S.E.M. of three independent experiments. Significant differences * *p* < 0.05; ** *p* < 0.01 express WT vs. knocked-out cells. (**B**) Graph summarizing results of PI exclusion assays.

The results of the PI exclusion assay confirmed our previous findings that the Doxo (0.5 µM; 1 µM) effect is significantly lower in the Jurkat CASP3/7/6^-/-^ and FADD^-/-^ cell lines compared to the WT line. The Jurkat WT mortality rate was 80%, in contrast to CASP3/7/6^-/-^ and FADD^-/-^ cells, which had a mortality range of 11–15%. This result confirms the importance of executioner caspases 3, 7, 6, and/or FADD protein in the initiation and execution of cell death upon treatment with Doxo (Figure 1).

In contrast to Doxo, the alkaloids efficiently induced the death of resistant cells. Upon SA (1 µg/mL) treatment, 80–95% of all Jurkat WT and FADD^-/-^ and CASP3/7/6^-/-^ cells died. A similar effect was observed upon treatment with SR (1 µg/mL and 2 µg/mL). SL (1 µg/mL) was also toxic to all cell lines; however, its toxicity was slightly less to FADD^-/-^ (50.4% dead cells) and CASP3/7/6^-/-^ (38.3% dead cells) cells in comparison to WT cells (75.8% dead cells). The effect of CHL (1 µg/mL) on all cell lines was similar, and mortality ranged from 58–73%. A higher concentration of CHL (2 µg/mL) was more toxic on WT and FADD^-/-^ cells (81–94% dead cells), while the effect on CASP3/7/6^-/-^ was lower. The most significant differences in the effect on WT and resistant cell lines were observed in the case of CHR (1 µg/mL). While in WT cells, 38% of cells died, in CASP3/7/6^-/-^ and FADD^-/-^ only 8% and 14% of cells died. However, upon a higher concentration of CHR (2 µg/mL), the mortality of the deficient cell lines was comparable with WT (about 73%), indicating the induction of necrosis. Interesting results were obtained with CHE (1 µg/mL), wherein a higher number of FADD^-/-^ cells died (81% dead cells), while a lower number of CASP3/7/6^-/-^ cells died (13% dead cells). Concerning WT, 45% of cells died.

### 2.2. Long-Term Effect of QBAs

**Figure 2:**
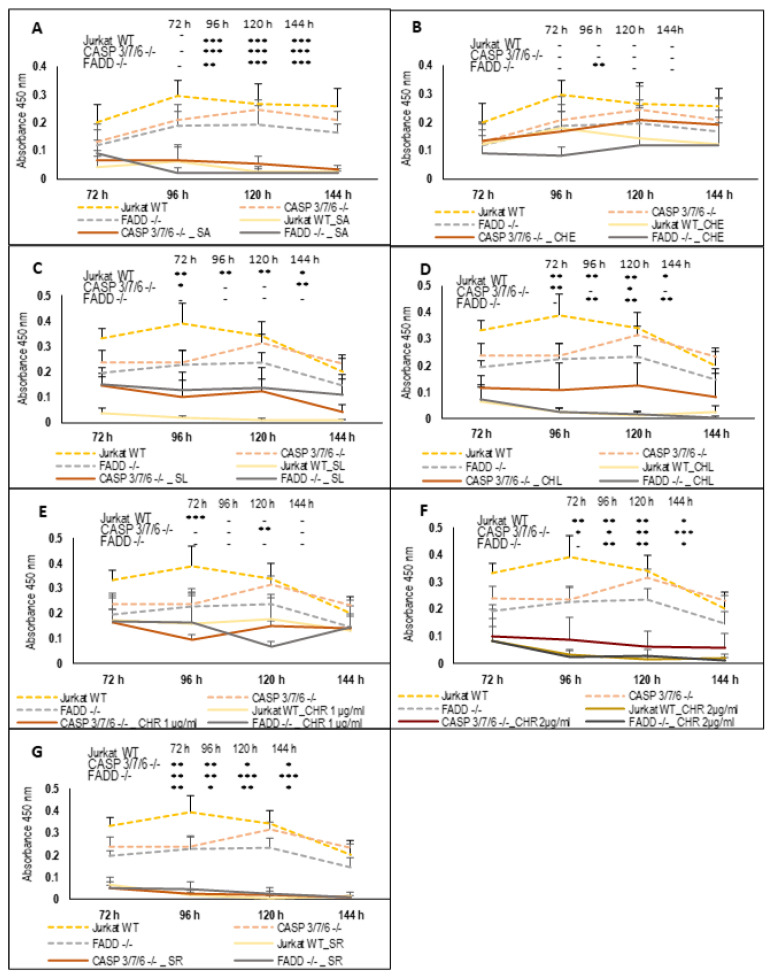
Long-term effect of QBAs on Jurkat WT, FADD^-/-^, and CASP3/7/6^-/-^ cells. Graphs representing results of WST metabolic activity assay. The effect of (**A**) SA; (**B**) CHE; (**C**) SL; (**D**) CHL, all at a concentration of 1 μg/mL, (**E**) CHR at concentrations of 1 μg/mL; (**F**) CHR at concentrations 2 μg/mL; and (**G**) SR at concentrations 1 μg/mL. The cells were incubated with alkaloids for 72 h and then medium was replaced with fresh medium containing drugs and incubated for additional hours (total time of incubation was 144 h). Data are expressed as absorbance values, which are proportional to the intensity of metabolic activity. Data are means ± S.E.M. of three independent experiments. Significant differences * *p* < 0.05; ** *p* < 0.01; *** *p* < 0.001 express untreated control vs. drug-treated cells.

We studied the long-term effect (72; 96; 120; and 144 h) of QBAs on Jurkat WT, FADD^-/-^, and CASP3/7/6^-/-^ cells by using the WST assay. The assay is based on the reduction of WST-1 by dehydrogenases in viable cells. This activity is directly proportional to the number of living cells. We incubated cells with QBAs for 72 h, then replaced the medium with fresh medium containing alkaloids, and incubated cells for additional hours (total 144 h). In control untreated cells, the amount of viable metabolically active cells decreased over time. The cells likely entered the plateau phase and stopped division after four days of cultivation (96 h) in response to their high density (Figure 2).

The results of the WST assay confirmed the results of the PI exclusion assay. SA stopped the proliferation of all cell lines, the CASP3/7/6^-/-^ response was slower, and cells died after prolonged exposure. The toxicity of CHE was low, and statistically significant only on FADD^-/-^ cells. FADD^-/-^ cells died more and CASP3/7/6^-/-^ less than WT, confirming the results of the PI-exclusion assay. SL (1 μg/mL) decreased metabolic activity in all cell lines over time, but the effect was lower on deficient CASP3/7/6^-/-^ and FADD^-/-^ cells. Jurkat WT cells revealed very low values of absorbance, which shows the absence of living cells. Compared to WT, SL-treated FADD^-/-^ and CASP3/7/6^-/-^ cells revealed higher metabolic activity which indicates partial resistance of deficient cells to SL. CASP3/7/6^-/-^ cells revealed a significant viability decrease after 144 h treatment. CHL (1 μg/mL) during this time significantly reduced the metabolic activity of Jurkat WT and FADD^-/-^ cells. After 96–120 h, all cells were probably dead. The effect of CHL on CASP3/7/6^-/-^ cells was highly variable, but it is apparent that CASP3/7/6^-/-^ cells were more resistant and revealed higher metabolic activity. CHR (1 μg/mL) slightly reduced the metabolic activity of cells of all cell lines. CHR at a concentration of 2 μg/mL showed a significantly decreased metabolic activity of all cells depending on the time of exposure. The effect on the Jurkat WT and FADD^-/-^ did not differ significantly, while CASP3/7/6^-/-^ cells revealed slightly higher metabolic activity. The effect of SR (1 μg/mL) was the same on all cell lines, and all cells were probably dead after 72 h. This is consistent with the result of the PI exclusion assay, which shows dead cells even after 48 h. The result suggests that the effect of SR does not depend on the action of FADD protein and/or executioner caspases.

The results of both PI exclusion and WST assays show that QBAs partially or completely overcome the resistance of CASP3/7/6^-/-^ and FADD^-/-^ cells. Upon SA and SR treatment, the percentage of dead cells in resistant cell lines was comparable to WT. Upon SL and CHL treatment, resistant cells died, but less than WT cells. CHR at a concentration of 1 μg/mL revealed low toxicity. However, at a higher CHR concentration (2 µg/mL), cells of all cell lines were dying, probably by necrosis. CASP3/7/6^-/-^ died the least upon CHE, CHL, and CHR treatment, suggesting the induction of caspase-dependent death, apoptosis. CHE affected the FADD^-/-^ cells the most.

### 2.3. QBAs Trigger Bimodal Cell Death

**Figure 3:**
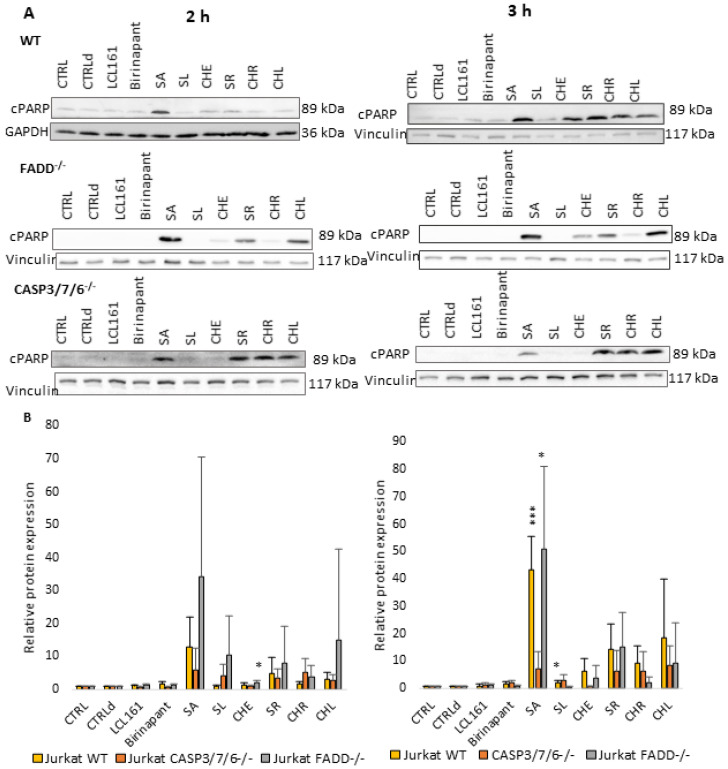
Western blot analysis of marker of apoptosis (cleaved PARP; cPARP). GAPDH and Vinculin were used as loading controls. (**A**) c-PARP detection after 2 h and 3 h treatment of WT, FADD^-/-^, and CASP3/7/6^-/-^ cells with QBAs at a concentration of 1 μg/mL. Total cell lysates were separated on 10% gels. (**B**) Graphs showing numeric values, which represent the ratio of band densities of cPARP normalized to the corresponding loading control and the untreated or DMSO-treated (CTLRd) control normalized to the corresponding loading control. Data are means ± S.E.M. of three independent experiments. Significant differences * *p* < 0.05; *** *p* < 0.001 express untreated control vs. drug-treated cells. CTRLd was used for LCL161 and Birinapant, which were dissolved in DMSO.

**Figure 4:**
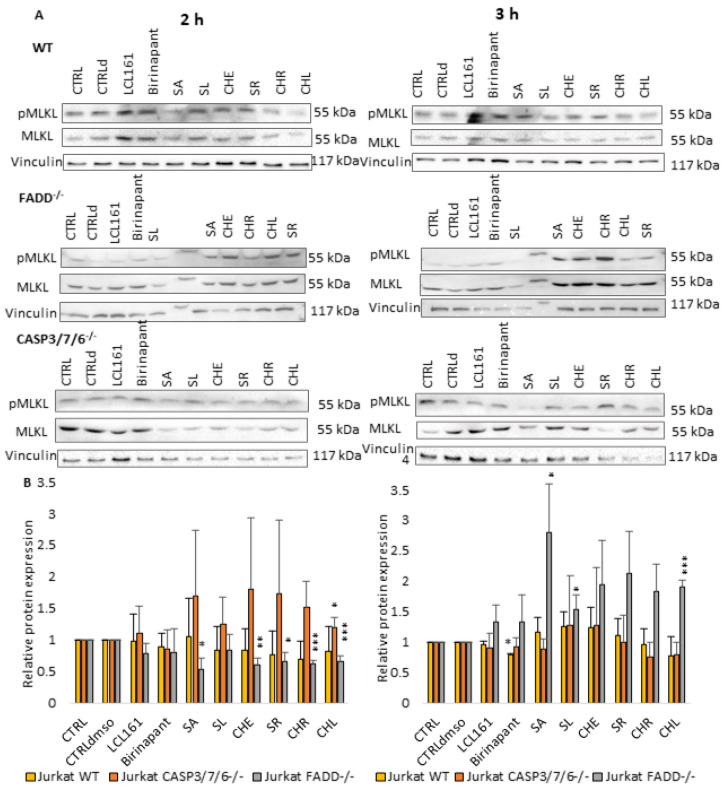
Western blot analysis of marker of necroptosis (pMLKL, MLKL). Vinculin was used as loading controls. (**A**) pMLKL, MLKL detection after 2 h and 3 h treatment of WT, FADD^-/-^, and CASP3/7/6^-/-^ cells with QBAs at a concentration of 1 μg/mL. Total cell lysates were separated on 10% gels. (**B**) Graphs showing numeric values, which represent the ratio of band densities of pMLKL to MLKL. Data are means ± S.E.M. of three independent experiments. Significant differences * *p* < 0.05; ** *p* < 0.01; *** *p* < 0.001 express untreated control vs. drug-treated cells. CTRLd (DMSO control) was used for LCL161 and Birinapant, which were dissolved in DMSO.

We tried to find the mechanism by which QBAs trigger cell death. We used Western blot analysis of the markers of apoptosis, cPARP (cleaved PARP, Figure 3), and necroptosis pMLKL (phosphorylated MLKL protein, Figure 4), after 2 and 3 h of incubation of Jurkat cells with QBAs. Smac mimetics, Birinapant (500 nM) and LCL161 (10 µM), were used as positive controls.

While SMs had little or no effect on PARP protein, the intensity of PARP cleavage upon individual alkaloids varied between the cell lines. Upon the effect of QBAs, PARP was cleaved the most in FADD^-/-^ cells and the least in CASP3/7/6^-/-^ cells. The effect of SA was the most prominent. FADD^-/-^ cells showed the most significant increase in cPARP upon the effect of all alkaloids. SA and CHL revealed the most pronounced effect. WT showed the most significant cleavage after 2 h treatment with SA and SR. CASP3/7/6^-/-^ cells responded the least. After 3 h, the cell response was similar to the 2 h treatment with SA showing the most pronounced effect on all cell lines. FADD^-/-^ cells showed weak responses to SL and CHR (Figure 3).

The necroptosis marker pMLKL was not significantly affected by SMs in any cells. QBAs after 2 h induced MLKL phosphorylation mainly in CASP3/7/6^-/-^ and WT, while in FADD^-/-^ cells the level of pMLKL was even lower compared to the control. In contrast, FADD^-/-^ cells revealed the most prominent increase in pMLKL after 3 h treatment with all QBAs. SA had the most pronounced effect. This indicates that necroptosis is triggered, but it is triggered later in FADD^-/-^ cells (Figure 4).

The results collectively show that cells trigger both apoptosis and necroptosis upon treatment with QBAs. Apoptosis is probably triggered first and is more prominent. CASP3/7/6^-/-^ cells exhibited a very low level of cPARP, while the level of pMLKL increased after 2 h of treatment with QBAs. This result is consistent with the fact that CASP3/7/6^-/-^ cells do not have functional executioner caspases. Non-apoptotic cell death probably predominates in these cells.

### 2.4. QBAs Act as Smac Mimetics and Decrease cIAP Protein Levels

Considering the results of previous works [26,27], which demonstrated the importance of SMs in cell death of deficient cells, we also investigated the effect of QBAs on the levels of antiapoptotic proteins cIAPs. SMs Birinapant (500 nM) and LCL161 (10 µM) were used as positive controls.

**Figure 5:**
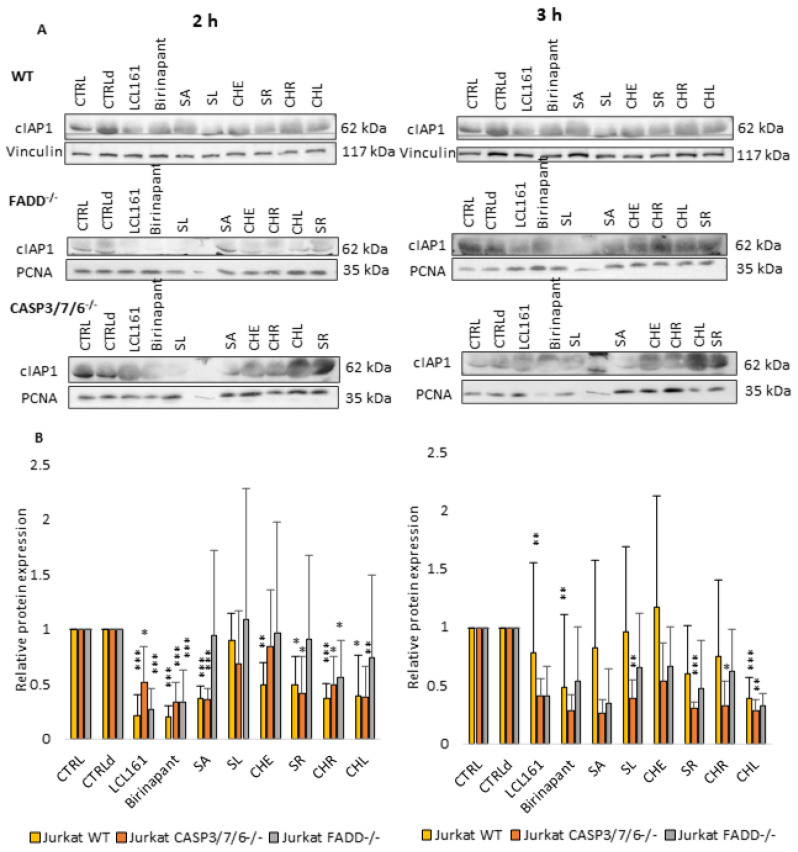
Western blot analysis of cIAP1 protein. Vinculin and PCNA were used as loading controls. (**A**) cIAP detection after 2 h and 3 h treatment of WT, FADD^-/-^, and CASP3/7/6^-/-^ cells with QBAs at a concentration of 1 μg/mL. Total cell lysates were separated on 10% gels. (**B**) Graphs showing numeric values, which represent the ratio of band densities of cIAP1 normalized to the corresponding loading control and the untreated or DMSO-treated (CTLRd) control normalized to the corresponding loading control. Data are means ± S.E.M. of three independent experiments. Significant differences * *p* < 0.05; ** *p* < 0.01; *** *p* < 0.001 express untreated control vs. drug-treated cells. CTRLd was used for LCL161 and Birinapant, which were dissolved in DMSO.

**Figure 6:**
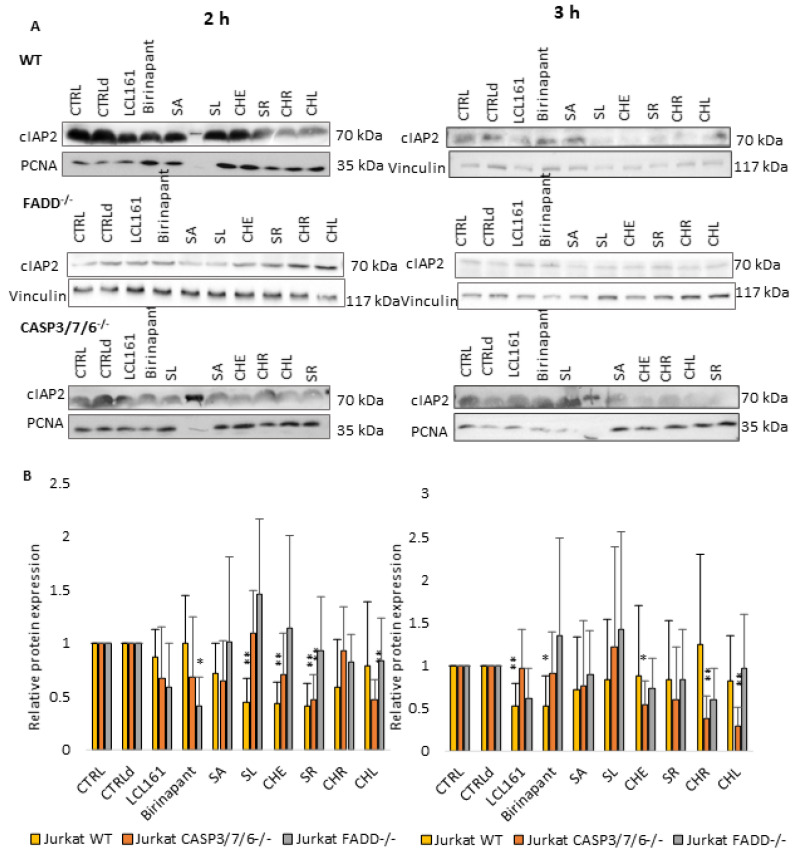
Western blot analysis of cIAP2 protein. Vinculin and PCNA were used as loading controls. (**A**) cIAP2 detection after 2 h and 3 h treatment of WT, FADD^-/-^ and CASP3/7/6^-/-^ cells with QBAs at a concentration of 1 μg/mL. Total cell lysates were separated on 10% gels. (**B**) Graphs showing numeric values, which represent the ratio of band densities of cIAP2 normalized to the corresponding loading control and the untreated or DMSO-treated (CTLRd) control normalized to the corresponding loading control. Data are means ± S.E.M. of three independent experiments. Significant differences * *p* < 0.05; ** *p* < 0.01 express untreated control vs. drug-treated cells. CTRLd was used for LCL161 and Birinapant, which were dissolved in DMSO.

Levels of both cIAP1 (Figure 5) and cIAP2 (Figure 6) proteins decreased upon QBAs and SMs treatment especially in WT and CASP3/7/6^-/-^ cell lines after 2 h of treatment. The decrease in the cIAP level was more pronounced in WT after 2 h, and in deficient cells after 3 h treatment. This indicates a slower response of deficient, especially FADD^-/-^ cells. SA revealed the most prominent effect, which was in the case of cIAP1 even comparable with SMs.

Taken together, it is probable that QBAs act similarly to SMs and decrease the levels of cIAP1 and cIAP2 proteins.

### 2.5. Effect of QBAs on the A375 Cell Line

We wondered whether alkaloids have similar effects on cells of solid tumors. In our previous work, the effect of the benzophenanthridine alkaloids SA, SL, CHE, CHL, and chelidonine on a malignant melanoma cell line (A375) was investigated. We demonstrated that individual QBAs, despite having very similar chemical structures, could induce different types of cell death. Most alkaloids were found to induce caspase-dependent cell death, apoptosis, while SL caused caspase-independent death, inhibited by necrostatin, presumably necroptosis [22,23].

In this work, we aimed to study in more detail by which mechanism the cell death in A375 cells is triggered. We again used Western blot analysis to detect markers of apoptosis (cPARP) and necroptosis (pMLKL) and levels of cIAP proteins in the A375 cell line. The design of the experiments was the same as on Jurkat cells.

**Figure 7:**
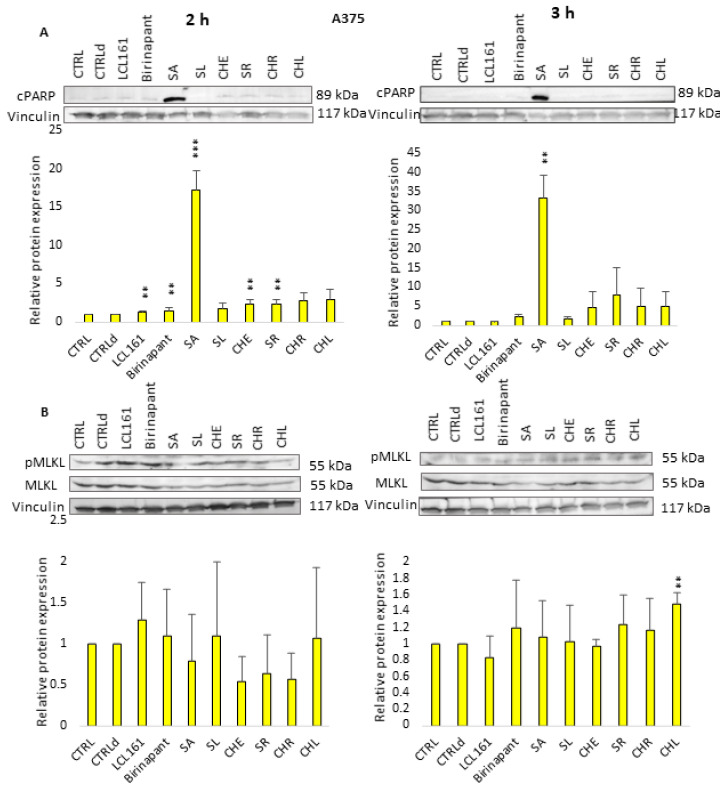
Western blot analysis of markers of apoptosis (cleaved PARP; cPARP) and necroptosis (pMLKL, MLKL). Vinculin was used as a loading control. (**A**) cPARP detection after 2 h and 3 h treatment of A375 cells with QBAs at a concentration of 1 μg/mL. Total cell lysates were separated on 10% gels. Graphs showing numeric values, which represent the ratio of band densities of cPARP normalized to the vinculin and the untreated or DMSO-treated (CTLRd) control normalized to vinculin. (**B**) pMLKL, MLKL detection after 2 h and 3 h treatment of A375 cells with QBAs at a concentration of 1 μg/mL. Total cell lysates were separated on 10% gels. Graphs showing numeric values, which represent the ratio of band densities of pMLKL to MLKL. Data are means ± S.E.M. of three independent experiments. Significant differences ** *p* < 0.01; *** *p* < 0.001 untreated control vs. drug-treated cells. CTRLd was used for LCL161 and Birinapant, which were dissolved in DMSO.

After 2 h and 3 h of A375 cells incubation with all QBAs, the cPARP protein was detected. SA revealed the most significant effect. The levels of pMLKL were not significantly increased or even decreased after 2 h treatment, but they were slightly increased after 3 h treatment with SR, CHR, and CHL. CHL had the most significant effect (Figure 7). It is possible that A375 melanoma cells are less sensitive to treatment and their response is weaker and slower than in Jurkat cells.

**Figure 8:**
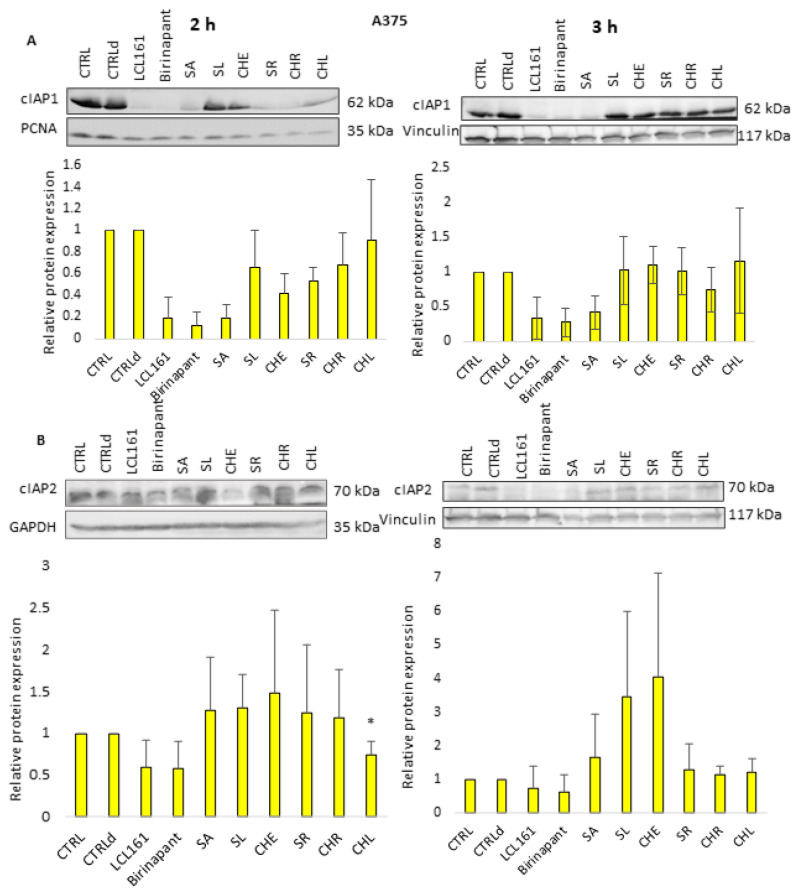
Western blot analysis of (**A**) cIAP1 and (**B**) cIAP2 proteins. GAPDH, Vinculin, and PCNA were used as loading controls. cIAPs detection after 2 h and 3 h treatment of A375 cells with QBAs at a concentration of 1 μg/mL. Total cell lysates were separated on 10% gels. Graphs showing numeric values, which represent the ratio of band densities of cIAP1 and/or cIAP2 normalized to the corresponding loading control and the untreated or DMSO-treated (CTLRd) control normalized to the corresponding loading control. Data are means ± S.E.M. of three independent experiments. Significant differences * *p* < 0.05 express untreated control vs. drug-treated cells. CTRLd was used for LCL161 and Birinapant, which were dissolved in DMSO.

The response of individual cIAP proteins in A375 cells was different. We observed the lowering of cIAP1 level more significantly after 2 h treatment with SMs and to a lesser extent with QBAs. The effect of SA was the most prominent. After 3 h treatment cIAP1 level was decreased only upon treatment with SMs, SA, and CHR. The response of the cIAP2 protein was much less pronounced. It showed a decrease in both times only after treatment with SMs. Interestingly, there was an increase in cIAP2 level, especially after 3 h treatment with SA, SL, and CHE (Figure 8).

## 3. Discussion

In the present study, we identified the mechanism of how QBAs can induce cell death of cancer cells resistant to apoptosis. Resistance to apoptosis is one of the hallmarks of cancer cells and represents a major obstacle in cancer treatment [28]. In our previous study investigating the effect of common anti-cancer drugs on Jurkat cells with knocked-out genes for key players in apoptosis and necroptosis, we revealed the critical importance of FADD protein and executioner caspases in the progression of programmed cell death. We found that the resistance of Jurkat FADD^-/-^ and CASP3/7/6^-/-^ cells could be overcome by treatment with a combination of TNF-α and SM (LCL161). This induced RIPK1-dependent cell death, necroptosis [27]. We wondered whether other agents could overcome the resistance. Since we have previously described interesting properties of QBAs [21,22,23,24,25] we focused on studying the effect of these natural products on resistant Jurkat cell lines.

Our results show that QBAs overcome the resistance of FADD^-/-^ and CASP3/7/6^-/-^ cells. Two of them, SA and SR revealed a comparable percentage of dead cells in WT and deficient cell lines. SL and CHL had a milder effect on the deficient cells. CHR showed the most significant difference in the effect on WT and deficient lines. By investigating markers of apoptosis and necroptosis cPARP and/or pMLKL, we examined the type of cell death triggered by QBAs. SA, SR, CHR, and CHL were the most important inducers of apoptosis, showing an ability to cleave PARP in WT and FADD^-/-^, and SA also in A375 cells. PARP cleavage in the CASP3/7/6^-/-^ cells was weak, which corresponds with the deficiency of effector caspases in this cell line. QBAs also increased the phosphorylation of MLKL, a marker of necroptosis. This was most prominent in CASP3/7/6^-/-^ cells after 2 h treatment and FADD^-/-^ after 3 h treatment. It is possible that QBAs primarily trigger apoptosis, but if the apoptosis pathways are disrupted, necroptosis can be triggered.

To find out if QBAs act in a similar way to SMs, we studied their effect on cIAP1 and cIAP2 proteins. In general, Western blot analysis showed the lowered levels of both cIAP proteins upon QBAs treatment in all cell lines. However, the response of FADD^-/-^ cells was only mild and was more significant for cIAP1 protein. The level of cIAP2 was almost unchanged or even slightly increased in FADD^-/-^ cells.

To test whether the effect of QBAs is the same for solid tumor cells, we investigated cIAPs levels after alkaloid treatment in human melanoma cells (A375). In A375 cells, all alkaloids decreased the level of cIAP1 after 2 h. The level of cIAP2 was decreased after 2 h only upon treatment with CHL, while treatment with other alkaloids especially after 3 h increased the cIAP2 level. An initial decrease and then increase in cIAP2 protein levels after the treatment with SMs was observed by Petersen et al. [7]. They detected an increase in cIAP2 level after cIAP1 knockdown. Similarly, Dardling et al. [29] proved that SM-mediated degradation of cIAP1 causes induction of cIAP2 gene expression, through the non-canonical activation of NF-kB. Therefore, in the absence of cIAP1, de novo synthesized cIAP2 can trigger resistance to SMs. These observations illustrate the complexity of the regulation of cellular signaling pathways and the causes of variability in cell responses.

**Figure 9:**
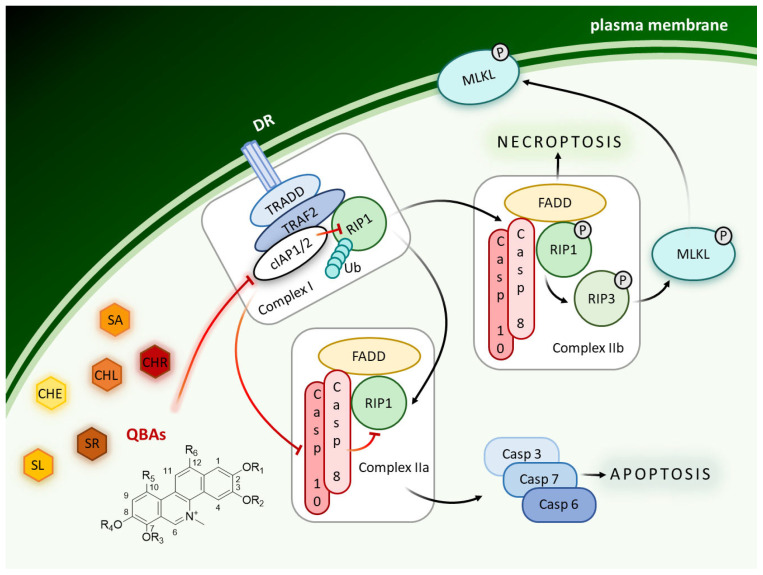
Graphical model demonstrating the mechanism of action of alkaloids.

It is probable that in both these cell lines, Jurkat and A375, both cell deaths are induced, with apoptosis apparently predominating and being triggered first (Figure 9). In A375 cells the response was slower. The alkaloids were less toxic to these cells. For SL, the apoptotic marker in A375 cells was only slightly apparent, confirming our earlier conclusion that SL induces necroptosis in A375 cells [23].

Several authors described the ability of SA and/or CHE to induce apoptosis. However, the mechanism of their action was not exactly clarified. The effect of QBAs, due to their chemical structure (planar molecule, iminium bond), is multitargeted [9,10,11,30,31]. They can interact with various cellular structures including DNA [9,32]. The induction of apoptosis and necrosis by SA was described by several authors. For a review, see Galadari et al. [30]. The ability of SA to reduce cIAP1, cIAP2, and XIAP protein levels has been described previously [33]. We previously detected a reduction in XIAP levels in the A375 cell line following the treatment with SA, CHE, SL, and CHL [22]. In the present work, we noted an interesting effect of CHE, namely a more pronounced toxicity on FADD^-/-^ than on WT cells. CASP3/7/6^-/-^ cells died the least. This suggests that CHE appears to predominantly induce apoptosis and targets more efficiently cells with FADD protein defects. Cheng et al. [34] found that FADD is indirectly involved in PKC (protein kinase C) inactivation by activating the catalytic subunit of the PP2A protease, which dephosphorylates and inactivates PKC. They observed that both FADD deficiency and the S191D mutant enhanced PKC phosphorylation, stability, and signaling. PKC signaling promotes proliferation and antagonizes cell death. Thus, it is possible that increased PKC activity contributes to the resistance of FADD^-/-^ cells and it could be the reason why CHE, as a PKC inhibitor [14], kills FADD^-/-^ cells more efficiently.

Collectively, our results show lowered levels of cIAPs and the presence of cPARP and pMLKL upon QBAs treatment. QBAs probably induce bimodal cell death, which has been described in Jurkat and patient samples after SMs treatment and after the treatment with cytostatics [26]. As mentioned above, only SA and CHE have been extensively studied, while the mechanism of action of other alkaloids studied in this work is unknown. The present study is the first that describes the ability of SL, CHL, CHR, and SR to affect the cIAP proteins levels.

## 4. Materials and Methods

### 4.1. Reagents

TNF-α, propidium iodide, BSA, and Immobilon Western Chemiluminescent HRP Substrate were purchased from Sigma-Aldrich (Darmstadt, Germany), WST-(2-(4-iodophenyl)-3-(4-nitrophenyl)-5-(2,4-disulfophenyl)-2H-tetrazolium, Na salt) from Serva (Heidelberg, Germany), and doxorubicin, Birinapant, and LCL161 from MedChemExpress (South Brunswick, NJ, USA).

### 4.2. Cell Lines and Cultivation Conditions

Jurkat WT cells and the knocked-out lines derived from them, CASP3/7/6^-/-^ and FADD^-/-^, were obtained from the laboratory of Dr. Bornhauser at the University Children’s Hospital Zurich. The lines were established using multicolor lentiCRISPR plasmids as described previously [26]. The A375 (human malignant melanoma) cell line was purchased from the European Collection of Animal Culture (ECACC, Salisbury, UK). The cells were grown in RPMI 1640 medium supplemented with 2 mM glutamine and 10% fetal bovine serum, 100 IU/mL penicillin, and 100 µg/mL streptomycin (Biosera, Cholet, France). Cells were incubated at 37 °C under 5% CO_2_ in a high-humidity atmosphere and subcultured three times a week.

### 4.3. Alkaloids

**Figure 10:**
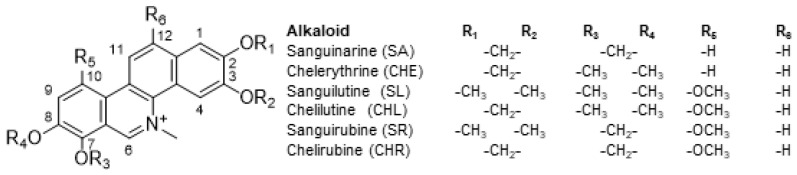
Quaternary benzophenanthridine alkaloids.

Sanguinarine chloride (SA), chelerythrine chloride (CHE), sanguirubine chloride (SR), chelirubine chloride (CHR), sanguilutine chloride (SL), and chelilutine chloride (CHL) were isolated from plants of the *Papaveraceae* family by Prof. J. Slavik and Prof. E. Táborská (Masaryk University Brno), who kindly provided them for our experiments [35,36,37] (Figure 10). Alkaloids were dissolved in distilled water at a concentration of 1 mg/mL (stock solutions) and stored at −20 °C.

### 4.4. Propidium Iodide Exclusion Assay

The cell viability assay was based on the exclusion of propidium iodide (PI) by the intact viable cells according to the protocol described in [27]. The cells were plated in 24-well tissue culture test plates (Techno Plastic Products, Trasadingen, Switzerland) at 5 × 10^4^ cells/mL and treated with QBAs for 48 h. After incubation, PI (1 μg/mL) was added and the percentage of dead (PI-positive) cells was detected using Cytomics FC 500 and/or CytoFlex flow cytometry systems (Beckman Coulter, Inc., Brea, CA, USA). A total of 10,000 cells were analyzed for each sample. Each experiment included two replicate wells and was repeated at least three times.

### 4.5. WST Assay

Antiproliferative activity was assessed using a WST assay on 96-well plates (Nunc A/S, Rockilde, Denmark) as was described in [38]. Briefly, the cells were seeded at a density of 5 × 10^4^ cells per mL (100 µL/per well) and incubated at 37 °C in a medium containing QBAs for 72 h. Then the medium was replaced with fresh medium containing alkaloid, and incubated for additional hours. After incubation (96; 120; 144 h), the WST assay was performed and optical density was read at 450 nm using an SLT Spectra-Shell-Microplate Reader (SLT-Lab Instruments GmBH, Saltsburg, Austria). Each concentration of each compound was examined in four replicate wells. The experiment was repeated three times.

### 4.6. SDS-PAGE and Western Blot Analysis

Cells were seeded at the concentration of 5 × 10^4^ cells/mL and treated with tested compounds for 2 and 3 h. After the treatment, cells were lysed in 2× Laemmli buffer. Whole-cell lysates were separated by electrophoresis on 10% SDS-PAGE gels and proteins were transferred onto methanol pre-treated PVDF membranes (Thermo Scientific, Waltham, MA, USA). Membranes were blocked with 2% bovine serum albumin (BSA) in TBS (10 mM TRIS-HCl, 100 nM NaCl, and 0.05% Tween 20; pH = 7.4) for 1 h at room temperature and incubated with primary antibodies diluted in 1% BSA at 4 °C overnight, followed by 1 h incubation with secondary antibodies. Signal was developed with Immobilon Western Chemiluminescent HRP Substrate (Sigma-Aldrich) and detected using GBOX iCHemi XRQ (Syngene, Cambridge, UK). The following primary antibodies were used: rat anti-MLKL (MABC604, Merck, Darmstadt, Germany), rabbit anti-phospho MLKL (Thr357, ABC234, Merck, Darmstadt, Germany), rabbit anti-cIAP1 (D5G9, 7065, Cell Signaling Technology, Danvers, MA, US), rabbit anti-cIAP2 (Ab23423, Abcam, Cambridge, UK), rabbit anti-cPARP (D64E10, 5625, Cell Signaling Technology, Danvers, MA, US), mouse anti-GAPDH, mouse anti-vinculin (sc-47724 and sc-73614, Santa Cruz Biotechnology, Dallas, TX, USA), and mouse anti-PCNA kindly provided by RNDr. Bořivoj Vojtěšek, DrSc. (Masaryk Memorial Cancer Institute, Brno, Czech Republic). Secondary antibodies conjugated to horseradish peroxidase (HRP): mouse m-IgGκ BP-HRP (sc-516102), goat anti-rat IgG-HRP (sc-2006), or mouse anti-rabbit IgG-HRP (sc-2357, Santa Cruz Biotechnology, Dallas, TX, USA) were used. The density of bands was analyzed by ImageJ Fiji software (version 1,53q). Graphs showing numeric values, which represent the ratio of band densities of protein of interest normalized to the corresponding loading control and the control normalized to the corresponding loading control. Two negative controls were used: CTRL for water-soluble alkaloids and CTRLd for LCL161 and Birinapant, which were dissolved in dimethylsulfoxide (DMSO). The concentration of DMSO corresponded to the concentration added with LCL161 (0.2 µL/mL) because the concentration added with Birinapant was lower (0.05 µL/mL).

### 4.7. Statistical Analysis

At least three independent experiments were performed under identical conditions. Data are expressed as the means ± SD. Statistical analysis compared untreated controls with treatments with alkaloids and was performed using TIBCO Software Inc. (2020; Data Science Workbench, version 14). Results were analyzed using the Student’s *t*-test, significant differences: *p* < 0.05; *p* < 0.01; *p* < 0.001.

## 5. Conclusions

In this work, we studied the response of four cancer cell lines to six natural products, QBAs. We demonstrated the ability of QBAs to trigger cell death in cancer cells resistant to apoptosis and identified a novel mechanism of their action, affection of antiapoptotic proteins cIAP1 and cIAP2. SA and SR completely and CHE, SL, and CHL partially overcome the resistance of CASP3/7/6^-/-^ and FADD^-/-^ cells. The results show that QBAs are able to induce bimodal cell death, i.e., both apoptosis and necroptosis. Apoptosis is probably triggered first and predominates. The response of WT cells in which cell death pathways are fully active is faster, in deficient cells especially in CASP3/7/6^-/-^ necroptosis probably predominates.

## Data Availability

All data are in the manuscript. All the original Western blot images are available in the editorial office or on request from corresponding author.
